# The Prevalence and Genetic Spectrum of Familial Hypercholesterolemia in Qatar Based on Whole Genome Sequencing of 14,000 Subjects

**DOI:** 10.3389/fgene.2022.927504

**Published:** 2022-07-15

**Authors:** Ilhame Diboun, Yasser Al-Sarraj, Salman M. Toor, Shaban Mohammed, Nadeem Qureshi, Moza S. H. Al Hail, Amin Jayyousi, Jassim Al Suwaidi, Omar M. E. Albagha

**Affiliations:** ^1^ College of Health and Life Sciences (CHLS), Hamad Bin Khalifa University (HBKU), Qatar Foundation (QF), Doha, Qatar; ^2^ Medical and Population Genomics Lab, Sidra Medicine, Doha, Qatar; ^3^ Qatar Genome Program, Qatar Foundation Research, Development and Innovation, Qatar Foundation (QF), Doha, Qatar; ^4^ Department of Pharmacy, Hamad Medical Corporation, Doha, Qatar; ^5^ Primary Care Stratified Medicine Research Group, Centre for Academic Primary Care, School of Medicine, University of Nottingham, Nottingham, United Kingdom; ^6^ Department of Diabetes, Hamad Medical Corporation (HMC), Doha, Qatar; ^7^ Adult Cardiology, Heart Hospital, Hamad Medical Corporation (HMC), Doha, Qatar; ^8^ Centre for Genomic and Experimental Medicine, MRC Institute of Genetics and Cancer, University of Edinburgh, Edinburgh, United Kingdom

**Keywords:** dyslipidemias, hypercholesterolemia, familial hypercholesterolemia, monogenic, FH, LDL-C, LDLR

## Abstract

Familial hypercholesterolemia (FH) is an inherited disease characterized by reduced efficiency of low-density lipoprotein-cholesterol (LDL-C) removal from the blood and, consequently, an increased risk of life-threatening early cardiovascular complications. In Qatar, the prevalence of FH has not been determined and the disease, as in many countries, is largely underdiagnosed. In this study, we combined whole-genome sequencing data from the Qatar Genome Program with deep phenotype data from Qatar Biobank for 14,056 subjects to determine the genetic spectrum and estimate the prevalence of FH in Qatar. We used the Dutch Lipid Clinic Network (DLCN) as a diagnostic tool and scrutinized 11 FH-related genes for known *pathogenic* and *possibly pathogenic* mutations. Results revealed an estimated prevalence of 0.8% (1:125) for definite/probable cases of FH in the Qatari population. We detected 16 known *pathogenic/likely pathogenic* mutations in *LDLR* and one in *PCSK9;* all in a heterozygous state with high penetrance. The most common mutation was rs1064793799 (c.313+3A >C) followed by rs771019366 (p.Asp90Gly); both in *LDLR*. In addition, we identified 18 highly penetrant *possibly pathogenic* variants, of which 5 were Qatari-specific, in *LDLR*, *APOB*, *PCSK9* and *APOE*, which are predicted to be among the top 1% most deleterious mutations in the human genome but further validations are required to confirm their pathogenicity. We did not detect any homozygous FH or autosomal recessive mutations in our study cohort. This pioneering study provides a reliable estimate of FH prevalence in Qatar based on a significantly large population-based cohort, whilst uncovering the spectrum of genetic variants associated with FH.

## Introduction

Familial hypercholesterolemia (FH) is a common autosomal disease characterized by elevated levels of low-density lipoprotein cholesterol (LDL-C), leading to an increased risk of atherosclerosis and premature coronary heart disease (CHD) (Bouhairie and Goldberg, 2015). The prevalence of FH in Caucasian populations has been typically considered around 1:500 (Austin et al., 2004), but more recent estimates show around 1:310 prevalence, with up to a 20-fold higher prevalence in those with premature CHD (Beheshti et al., 2020; Hu et al., 2020). Indeed, a recent study based on 225 Chinese subjects with premature myocardial infarction revealed up to 23.6% prevalence of FH (Cui et al., 2019). Notably, FH has a strong genetic basis compounded by environmental factors and its prevalence varies amongst different populations. Homozygous FH (HoFH) is rare; typically considered to affect 1 in 1,000,000 worldwide, but recent reports have estimated a higher prevalence of 1 in 160,000–300,000 (Cuchel et al., 2014; Singh and Bittner, 2015; Sjouke et al., 2015). HoFH is characterized by a drastic increase in LDL-C (>13 mmol/L before therapy) and the early development of atherosclerotic complications (Cuchel et al., 2014). Heterozygous FH (HeFH) is more common with an estimated prevalence of 1 in 250 in European populations (Vrablik et al., 2020). However, variations in FH prevalence have been reported in certain populations such as 1 in 137 in the Danish population (Benn et al., 2012), 1 in 270 in the United Kingdom population (Wald et al., 2016), and 1 in 311 in the Russian population (Meshkov et al., 2021). Notably, FH is considerably higher in certain populations such as Ashkenazi Jews (1 in 67) or Christian Lebanese (1 in 85) and South African Afrikaners (1 in 72), attributing to the founder effect which contributes to the significantly higher incidence (Austin et al., 2004; Henderson et al., 2016).

Mutations in *LDLR*, *APOB* and *PCSK9* account for most FH cases and have been reported in both HoFH and HeFH. These three genes are members of the low-density lipoprotein receptor (LDL-R) pathway, which is responsible for maintaining healthy levels of plasma and intracellular cholesterol in the body. The vast majority of *LDLR* variants are deleterious (79%), affecting the splicing or regulatory aspects of early transcription or alternatively, leading to a defective receptor function (Henderson et al., 2016). Owing to the extensive spectrum of *LDLR* variants in FH, mutations in the *LDLR* gene can be population specific (Vrablik et al., 2020). Less stricter forms of FH have been associated with variants in *APOB*, a gene whose protein product helps LDL bind to its receptor LDL-R, but it is observed in about 5% of FH cases in European populations (Henderson et al., 2016; Vrablik et al., 2020). Mutations leading to a gain of function of *PCSK9*, an enzyme that promotes the degradation of LDL-R, could account for <1% of FH cases (Henderson et al., 2016). Less commonly observed variants of FH have been described in the *LDLRAP1* gene causing an autosomal recessive form of the disease termed autosomal recessive hypercholesterolemia (ARH) (Garcia et al., 2001). Interestingly, ARH is characterized with LDL-C levels in between the HeFH and HoFH known levels, and unlike HoFH, the early-onset phenotype is comparatively rare (Henderson et al., 2016). Further to monogenic variants, polygenic effects have also been described to account for hypercholesterolemia (HC) cases (Saadatagah et al., 2021). Notably, more than 900 lipid-associated genomic loci have been identified in genome-wide studies (GWAS) based on investigating genetic variants in diverse ancestries (Graham et al., 2021).

The most widely accepted guidelines for the diagnosis of FH include the Simon-Broome Register criteria (Mortality, 1999), the Dutch Lipid Clinic Network (DLCN) criteria (Umans-Eckenhausen et al., 2001) and the US MedPed program (Williams et al., 1993). The DLCN criteria is a comprehensive tool, which considers multiple parameters in the assessment of FH including patients’ personal and family history related to the onset of early cardiovascular (CVD) or HC, untreated LDL-C concentration, disease clinical manifestations (including tendinous xanthomas and arcus cornealis) and functional mutation in a pathogenic FH-related gene (Defesche et al., 2004). However, despite the comprehensiveness of available diagnostic schemes, FH remains largely underdiagnosed.

The prevalence of FH in Qatar is not yet known. However, FH prevalence (probable and definite cases) in neighboring Gulf countries (Saudi Arabia, Oman, United Arab Emirates, Bahrain and Kuwait) was estimated at 1:232 (Al-Rasadi et al., 2020) and subsequently stratified to fulfill the DLCN criteria to reveal a markedly higher prevalence of 1:112, but genetic screening was not performed in this study (Alhabib et al., 2021). The high burden of lipid disorders in the region has also led to continued recommendations to improve the management of lipid disorders (Alsayed et al., 2022). In addition, accumulating studies have also explored the genetic spectrum of FH in the wider Middle Eastern region (Awan et al., 2019), with reports of mutations occurring predominantly in *LDLR* (Alhababi and Zayed, 2018). Notably, a recent study reported mutations in 6 *LDLR* variants in a subset of ∼6,000 individuals from Qatar, but no phenotypic analyses or evidence were sought for FH (Elfatih et al., 2021). Moreover, GWAS have also linked specific lipid risk variants to various populations in the region (Hebbar et al., 2019), specifically in Qatar (Thareja et al., 2021). Therefore, comprehensive investigations into FH etiology in Qatar are warranted.

In this study, we estimated the prevalence of FH in Qatar, while uncovering the mutational spectrum and penetrance of population-specific FH-related monogenic variants using whole-genome sequencing of the population-based cohort of Qatar biobank (QBB; *n* = 13,808). We sought to decipher mutations in genes previously linked with FH pathogenicity and identify highly penetrant Qatari-specific variants. Our findings have the potential to empower the current diagnostic approaches by allowing targeted genetic screening of high-risk individuals and cascade screening of suspected FH cases.

## Materials and Methods

### Study Populations

This study was based on 14,056 Qatari subjects from the population-based Qatar Biobank (QBB) study (Al Kuwari et al., 2015) and was executed under ethical approvals from the institutional review boards of QBB, Doha, Qatar (Protocol no. E-2020-QF-QBB-RES-ACC-0154-0133) and Hamad Bin Khalifa University (Approval no. 2021-03-081). All participants provided written informed consent prior to sample donation. Participants also underwent a medical examination and filled out an approved and standardized questionnaire, which captured information on medical history. The study cohort is deeply phenotyped, featuring lipid-related measurements including total cholesterol, HDL-C, LDL-C, and triglycerides, and detailed information on diagnosis, comorbidities related to heart disease and administration of cholesterol-lowering medication was also accessible. However, while information on the family history of HC was recorded, lipid measurements from close relatives were not available.

### Whole-Genome Sequencing

Whole-genome sequencing of the cohort was available through the Qatar Genome Program (QGP) and was performed as previously described (Mbarek et al., 2022). Briefly, libraries were constructed using the Illumina TruSeq DNA nano kit and indexed using Illumina TruSeq Single Indexes (Illumina, San Diego, CA, United States). Quality-passed libraries were sequenced on an Illumina HiSeq X instrument. The quality metrics for generated Fastq files were assessed using FastQC (v. 0.11.2). The raw reads were trimmed and aligned to hs37d5 reference genome using bwa.kit (v0.7.12) (Li and Durbin, 2009) to generate mapped reads on BAM files. The coverage of each sample was evaluated using Picard (v1.117) (CollectWgsMetrics), while variant calling was performed using base quality score recalibration (BQSR) and intermediate genomic gVCF (gVCF) was generated by running HaplotypeCaller (Genome Analysis Toolkit; GATK). The genotype data were processed for quality control using Hail (Team, 2022) and Plink (Slifer, 2018). Genetic variants with <98% call rate, chi-square test *p* value for Hardy-Weinberg equilibrium <1 × 10^−10^, or those with a depth of coverage <10X were removed, while only variants with minor allele frequency <0.005 were included in the analysis, calculated based on estimating FH prevalence of ∼1 in 200. Subjects with excess heterozygosity, gender ambiguity, or with call rate <95% were also excluded. The final study cohort comprised 13,808 subjects with both whole-genome sequence data and complete phenotype data.

### Familial Hypercholesterolemia Evaluation Criteria

The DLCN scheme draws evidence from high LDL-C levels, the presence of functional mutations in *LDLR*, *APOB* or *PCSK9* genes, xanthomas and corneal deposits of fat, and evidence of coronary and vascular disease in subjects and their close relatives for diagnosing FH (Austin et al., 2004). The assessment of the phenotypic or genetic parameters assigns numerical scores to classify subjects with unlikely FH (DLCN score: <3), possible FH (DLCN score: 3–5), probable FH (DLCN score: 6–8) or definite FH (DLCN score >8) (Austin et al., 2004). We followed these criteria to characterize FH cases, while performing correction of LDL-C levels for cholesterol-lowering medication as defined by Haralambos *et al.* (Haralambos et al., 2015). In instances where the medication dose was not available, we used the minimum dose for correction. Of note, the presence of xanthomas and corneal fat deposition was not recorded by QBB and these parameters were not taken into consideration in our study. Importantly, the remaining criteria were sufficient to classify patients into definite/probable/possible or unlikely FH cases based on DLCN scores.

### Variant Annotation and Assessment of Pathogenicity

We used the bcftools software (version 1.10.2) to extract records of variants located in selected FH genes from the variant calling format (VCF) genotype file. The selection of FH genes was based on a literature search and included *LDLR*, *APOB*, *PCSK9*, *APOE*, *LDLRAP1*, *CYP7A1*, *STAP1*, *ITIH4*, *EPHX2*, *GHR* and *PPP1R17* (also known as *GSBS*) genes (Takada et al., 2003; Fujita et al., 2004; Sato et al., 2004; Paththinige et al., 2017; Wang et al., 2020). Variant annotation was performed using SnpEff/SnpSift (v4.3t) (Cingolani et al., 2012) utilizing dbSNP build version 151 (Sherry et al., 1999), ClinVar (Landrum et al., 2018), Human Gene Mutation Database (HGMD) variant categorization (Stenson et al., 2020) and Leiden Open Variation Database (version: 3.0; LOVD3) (Fokkema et al., 2021). Variants were considered pathogenic for FH if reported as *pathogenic/likely pathogenic* or *disease-causing mutation (DM)* or *likely disease-causing mutation (DM?)* by at least two of the databases described earlier. We assessed the penetrance of genetic variants using two approaches; the first was based on the DLCN score (DLCN-penetrance), which takes into consideration multiple phenotypic variables related to FH risk as described earlier. Using this approach, penetrance was defined as the proportion of mutation carriers with a DLCN score ≥3. The second approach was based on LDL-C and self-reported HC (HC-penetrance) by which penetrance was defined as the proportion of mutation carriers with LDL-C > 3.3 mmol/L, or taking cholesterol-lowering medication, or self-reported HC. For variants of uncertain significance (VUS), we considered them *possibly pathogenic* if their HC-penetrance value was >50% and their predictive combined annotation dependent depletion (CADD) (Kircher et al., 2014) score was greater than 20, which represents the top 1% most deleterious mutations in the human genome.

### Statistical Analysis

The Hardy-Weinberg equilibrium of genotypes was evaluated by χ2 tests. Unpaired Student’s t test was used for comparison between individuals with definite, probable, and possible FH relative to those with unlikely FH. The prevalence of each definition of FH was estimated as a percentage for all study subjects. Fisher’s exact test was used to compare the frequency of factors among the definitions of FH. A gene-based burden test was performed using SAIGE-Gene (Zhou et al., 2020) by collapsing all *pathogenic*/*possibly pathogenic* variants detected in each gene into a single burden variable, and in this analysis model, we adjusted for age, sex, genomic kinship and the first four population principal components.

## Results

### Dutch Lipid Clinic Network Classification Based on Phenotypic Traits

**FIGURE 1:**
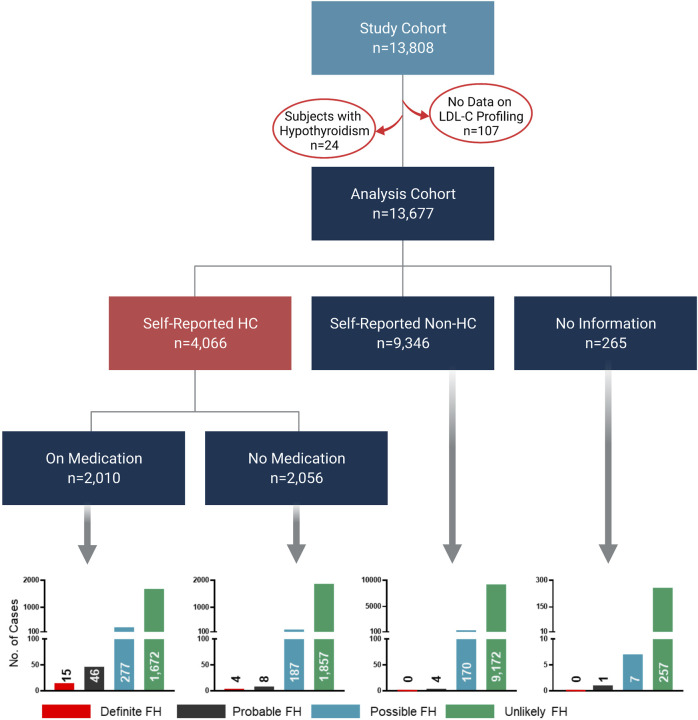
Classification of study cohort into FH subtypes based on phenotypic data. The flow chart depicts the workflow for the classification of study cohorts into FH subtypes based on phenotypic data only to fulfill DLCN criteria for FH classification. Bar charts represent numbers of individuals classified as definite, probable, possible and unlikely cases of FH in the analysis cohort.

This study was based on phenotypic and genotypic data from 13,808 individuals. However, the analysis cohort comprised 13,677 subjects since LDL-C profiling measurements were not available for 107 subjects, while additional 24 subjects with hypothyroidism were removed from the analysis (Figure 1). We first categorized our cohort based on self-reports of HC; 4,066 subjects declared HC, while 9,346 subjects reported no HC and the remaining 265 subjects did not provide any information. Moreover, among the self-reported HC subjects, 2,010 subjects declared taking cholesterol-lowering medication. Importantly, our initial classifications showed that 19 subjects were classified as definite cases of FH based on the fulfillment of the DLCN criteria (DLCN score >8) by phenotypic evidence only (Figure 1). Similarly, probable, possible and unlikely FH cases were also first classified based on phenotypic evidences only. The characteristic features of the overall study cohort classified into FH categories based on phenotypic evidence are listed in Table 1. Of note, the definite FH cases cohort comprised individuals with considerably higher percentages of self-reported HC and a history of paternal and maternal heart disease compared to those in the unlikely FH group (Table 1). Moreover, subjects in the definite FH group had a lower age of HC diagnosis (35.5 ± 11.6) compared to subjects in the unlikely FH category (40.6 ± 10.6), but this was of borderline significance (*p* = 0.06).

**TABLE 1:** Characteristics of Qatar Biobank study subjects according to the Dutch Lipid Clinic Network (DLCN) criteria based on phenotypic traits only.

Phenotypic trait(s)	Definite FH	Probable FH	Possible FH	Unlikely FH
Characteristic features				
n	19	59	641	12,958
Gender (males/females)	7/12	30/29	362/279	5,680/7,278
Age (years)	47.9 ± 12.2*	47.7 ± 11.9*	46.4 ± 11.6*	39.7 ± 13.1
BMI (kg/m^2^)	30.3 ± 4.5	30.0 ± 5.8	30.1 ± 5.4*	29.5 ± 6.1
Smoker (%)	6 (31.6%)	8 (13.6%)	145 (22.6%)*	2,175 (16.8%)
Medical history				
Hypertension (%)	4 (21.1%)	23 (39.0%)*	160 (25.0%)*	1989 (15.3%)
Diabetes mellitus (%)	9 (47.4%)*	30 (50.8%)*	197 (30.7%)*	2,563 (19.8%)
Self-reported hypercholesterolemia (HC)	19 (100%)*	53 (89.8%)*	450 (70.2%)*	3,438 (26.5%)
Age at HC diagnosis (years)	35.5 ± 11.6	38.3 ± 10.2	39.6 ± 10.4	40.6 ± 10.6
Cholesterol-lowering medication	15 (78.9%)*	46 (78.0%)*	277 (43.2%)*	1,672 (12.9%)
History of myocardial infarction (MI)	0 (0.0%)	3 (5.1%)*	31 (4.8%)*	35 (0.3%)
Age at MI (years)	n/a	37.3 ± 13.5	44.6 ± 8.0	49.5 ± 14.9
History of angina	0 (0.0%)	0 (0.0%)	21 (3.3%)*	24 (0.2%)
Paternal heart disease (%)	14 (73.7%)*	18 (30.5%)	168 (26.2%)*	2,760 (21.2%)
Maternal heart disease (%)	7 (36.8%)*	12 (20.3%)	109 (17.0%)*	1,615 (12.5%)
Lipid profile				
Total cholesterol (mmol/L)	8.35 ± 2.03*	7.16 ± 1.37*	6.41 ± 1.16*	4.82 ± 0.86
HDL-C (mmol/L)	1.36 ± 0.28	1.30 ± 0.34*	1.29 ± 0.37*	1.41 ± 0.39
LDL-C (mmol/L)	9.83 ± 1.01*	8.46 ± 1.88*	5.51 ± 0.93*	3.02 ± 0.82
Triglycerides (mmol/L)	2.16 ± 1.05*	1.81 ± 0.78*	1.72 ± 0.85*	1.27 ± 0.73
DLCN Score	9 ± 0*	6.8 ± 1.0*	3.5 ± 0.7*	0.4 ± 0.6

^†^Continuous traits are given as mean ± standard deviation from the mean.*Statistically significant (P <0.05) compared to unlikely FH.

### Familial Hypercholesterolemia Pathogenic Variants Detected in the Qatari Population

**FIGURE 2:**
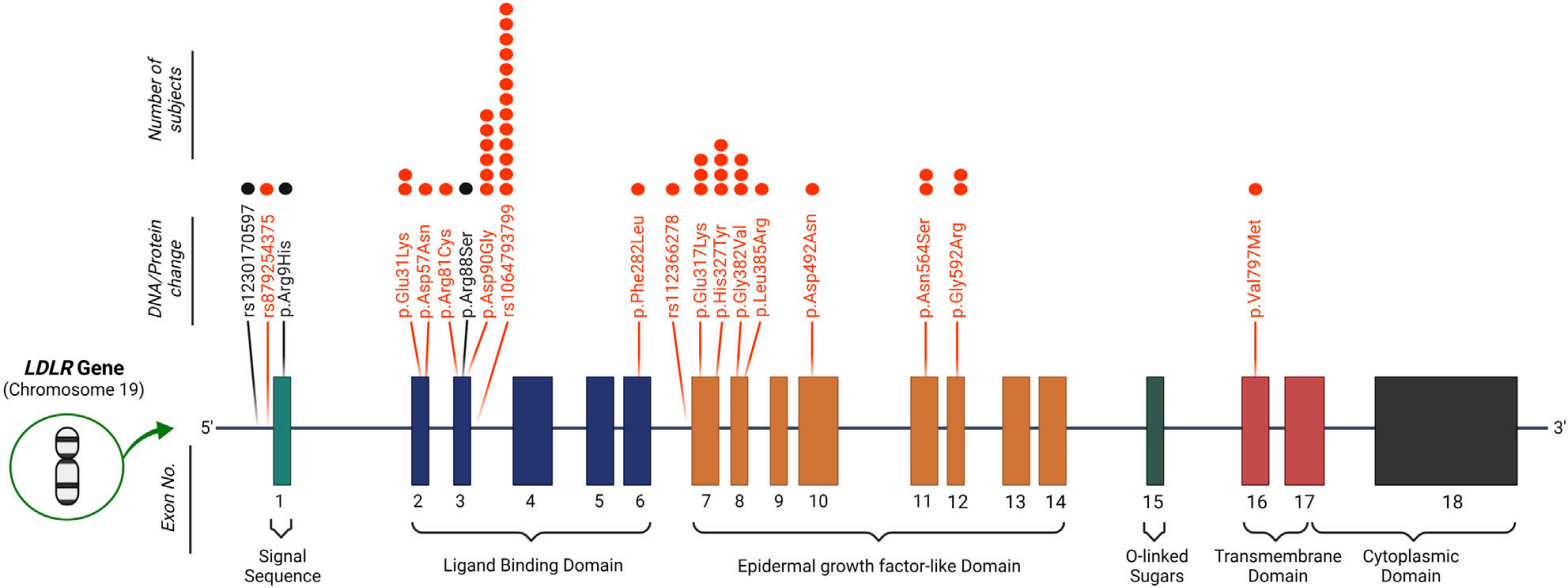
Identified mutations in the *LDLR* gene. 16 known *pathogenic* variants (marked in red) and 3 *possibly pathogenic* mutations (marked in black) were detected in *LDLR* in our study cohort. The number of subjects carrying each mutation is depicted by dots above each variant. Protein change is in reference to the NCBI sequence NP_000518.1.

Applying the DLCN criteria for FH diagnosis entails the identification of functional mutations in *LDLR*, *APOB* and *PCSK9* genes. To this end, variants were scrutinized for their pathogenicity using ClinVar, LOVD3 and HGMD significance terms to identify previously reported *pathogenic* variants in the Qatari population. Our search for variants covered 11 FH-related genes (refer to methods). We detected 17 variants that were reported as *pathogenic/likely pathogenic* or *DM/DM?* by at least two of the ClinVar/LOVD3/HGMD databases (Table 2); all as heterozygous. Notably, 16 variants were located in *LDLR* (Figure 2) with rs1064793799 (c.313+3A>C) being the most frequent mutation (*n* = 13), followed by rs771019366 (p.Asp90Gly; *n* = 6) and rs747507019 (p.His327Tyr; *n* = 4), while one mutation was located in *PCSK9* (rs891322948, *n* = 2). Moreover, the majority of these were missense mutations, which lead to specific protein changes while others were splice variants or associated with regulatory regions. Most mutations showed high DLCN-penetrance (>50%) and the majority had complete HC-penetrance (100%). We did not detect any previously reported *pathogenic* mutations in *APOB*, *APOE*, *LDLRAP1*, *CYP7A1*, *STAP1*, *ITIH4*, *EPHX2*, *GHR,* or *PPP1R17*.

**TABLE 2:** Known *pathogenic* variants detected in the study subjects.

Gene	SNP	Position	Ref	Alt	Protein change	No. Het	MAF	HGMD class.	LOVD class.	ClinVar sig.	DLCN-penetrance (%)	HC-penetrance* (%)
*LDLR*	rs879254375	chr19:11089414	C	G	TF-binding	1	3.41 × 10^−5^	DM	LP	VUS	100	100
*LDLR*	rs776421777	chr19:11100246	G	A	p.Glu31Lys	2	6.82 × 10^−5^	DM	LP	LP/VUS	0	100
*LDLR*	rs879254420	chr19:11100324	G	A	p.Asp57Asn	1	3.41 × 10^−5^	DM?	LP	P/LP/VUS	100	100
*LDLR*	rs730882078	chr19:11102714	C	T	p.Arg81Cys	1	3.41 × 10^−5^	DM	LP	NR	0	100
*LDLR*	rs771019366	chr19:11102742	A	G	p.Asp90Gly	6	2.05 × 10^−4^	DM	LP	P/LP	83.3	100
*LDLR*	rs1064793799	chr19:11102789	A	C	Splice variant	13	4.43 × 10^−4^	DM	NR	P	84.6	100
*LDLR*	rs730882090	chr19:11107420	C	A	p.Phe282Leu	1	3.41 × 10^−5^	DM	LP	P/LP/VUS	100	100
*LDLR*	rs112366278	chr19:11110650	A	C	Splice variant	1	3.41 × 10^−5^	DM	LP	P/LP	100	100
*LDLR*	rs746834464	chr19:11110660	G	A	p.Glu317Lys	3	1.02 × 10^−4^	DM?	LP	P/LP	0	33.3
*LDLR*	rs747507019	chr19:11110690	C	T	p.His327Tyr	4	1.36 × 10^−4^	DM	LP	VUS	25	100
*LDLR*	rs752951310	chr19:11111598	G	T	p.Gly382Val	3	1.02 × 10^−4^	DM	P/LP	P/LP/VUS	66.7	100
*LDLR*	rs879254809	chr19:11111607	T	G	p.Leu385Arg	1	3.41 × 10^−5^	DM	LP	LP	100	100
*LDLR*	rs373646964	chr19:11113650	G	A	p.Asp492Asn	1	3.41 × 10^−5^	DM	LP	NR	100	100
*LDLR*	rs758194385	chr19:11116198	A	G	p.Asn564Ser	2	6.82 × 10^−5^	DM	LP	P/LP	50	50
*LDLR*	rs763147599	chr19:11116927	G	A	p.Gly592Arg	2	6.82 × 10^−5^	DM	P	P/LP	50	50
*LDLR*	rs750518671	chr19:11128085	G	A	p.Val797Met	1	3.41 × 10^−5^	DM	LP	NR	100	100
*PCSK9*	rs891322948	chr1:55059529	G	T	p.Gly516Val	2	6.82 × 10^−5^	DM?	LP	NR	100	100

Alt, alternative allele; DM, disease-causing mutation; DM?, likely disease-causing mutation; FH, familial hypercholesterolemia; Het, heterozygous; HGMD, Human Gene Mutation Database; LP, likely pathogenic; LOVD, Leiden Open Variation Database; MAF, minor allele frequency; NR, not reported; P, pathogenic; Ref, reference allele; VUS, variant of uncertain significance; *HC, high cholesterol (refer to methods of details). Protein positions are in reference to the NCBI sequence NP_000518.1.

### Possibly Pathogenic Variants in the Qatari Population

We identified 18 *possibly pathogenic* variants as those which were predicted to be in the top 1% of most deleterious mutations in the human genome (CADD score >20) and their HC-penetrance is ≥50% (Table 3). These mutations were detected in FH-related genes including *LDLR*, *APOB*, *PCSK9* and *APOE*, and featured a wide range of deleterious effects, including stop-gained and missense, in addition to impacting regulatory features. Five of the 18 detected variants were novel since they were not reported in genetic databases such as dbSNP and the Genome Aggregation Database (gnomAD), and were considered to be Qatari-specific. The most common *possibly pathogenic* variants were located in *APOB* (rs775231207; *n* = 7, rs13306190; *n* = 4, and rs12713559; *n* = 4). Overall, the majority of mutations observed were missense, while one stop-gain was detected in *PCSK9*. Notably, rs1230170597 is located 169 bp upstream of the coding region of *LDLR* and overlaps *LDLR-AS1*, an antisense non-coding RNA predicted to downregulate production of the LDL-R. However, these *possibly pathogenic* mutations were not used in DLCN classification because functional validation will be required to confirm their pathogenicity. Gene-based burden test LDL-R for the *pathogenic* and *possibly pathogenic* variants detected in our cohort showed significant associations with HC for *LDLR* (*p* <0.0001; Beta (β) = 3.2; standard error (SE) = 0.52), *PCSK9* (*p* = 0.004; β = 2.6; SE = 1.08), and *APOB* (*p* = 0.004; β = 1.1; SE = 0.37) but not for *APOE* (*p* = 0.303). Genetic variations that fit the “*possibly pathogenic*” criteria were not detected in the other FH-related genes: *LDLRAP1*, *CYP7A1*, *STAP1*, *ITIH4*, *EPHX2*, *GHR*, or *PPP1R17.*

**TABLE 3:** *Possibly pathogenic* variants with high penetrance detected in the study subjects.

Gene	Variant ID	Position	Ref	Alt	Protein change	No. het	MAF	HGMD class.	ClinVar sig.	LOVD class.	HC-penetrance (%)	CADD score
*LDLR*	rs1230170597	chr19:11089380	G	C	Promoter	1	3.41 × 10^−5^	NR	VUS	NR	100	20.5
***LDLR***	**NR**	**chr19:11089574**	**G**	**A**	**p.Arg9His**	**1**	**3.41 × 10** ^**−5**^	**NR**	**NR**	**NR**	**100**	**22.8**
*LDLR*	rs879254454	chr19:11102737	G	T	p.Arg88Ser	1	3.41 × 10^−5^	NR	VUS	NR	100	21
*APOB*	rs267599185	chr2:21013214	G	A	p.Arg1388Cys	2	6.82 × 10^−5^	NR	VUS	LB	50	20.7
***APOB***	**NR**	**chr2:21006246**	**A**	**G**	**p.Ile3541Thr**	**2**	**6.82 × 10** ^**−5**^	**NR**	**NR**	**NR**	**50**	**23.3**
***APOB***	**NR**	**chr2:21008255**	**A**	**T**	**p.Asn2871Lys**	**1**	**3.41 × 10** ^**−5**^	**NR**	**NR**	**NR**	**100**	**22**
*APOB*	rs1440306074	chr2:21012016	A	T	p.Ser1618Thr	1	3.41 × 10^−5^	NR	NR	NR	100	22.8
*APOB*	rs775231207	chr2:21012456	A	C	p.Leu1471Trp	7	2.30 × 10^−4^	NR	NR	NR	57	21.8
*APOB*	rs1208454201	chr2:21012474	A	G	p.Met1465Thr	2	6.82 × 10^−5^	NR	NR	NR	50	21
*APOB*	rs140877474	chr2:21012493	T	C	p.Ser1459Gly	2	6.82 × 10^−5^	NR	VUS	NR	50	25.5
***APOB***	**NR**	**chr2:21015242**	**A**	**G**	**p.Phe1176Ser**	**1**	**3.41 × 10** ^**−5**^	**NR**	**NR**	**NR**	**100**	**32**
*APOB*	rs765952330	chr2:21015514	C	G	p.Gly1122Arg	2	6.82 × 10^−5^	NR	NR	NR	50	25.2
*APOB*	rs781511068	chr2:21023005	G	A	p.Val881Ala	2	6.82 × 10^−5^	NR	VUS	NR	50	27
*APOB*	rs13306190	chr2:21032408	G	A	p.Ala433Val	4	1.36 × 10^−4^	NR	LB	NR	50	24.9
*APOB*	rs12713559	chr2:21006196	G	A	p.Arg3558Cys	4	1.36 × 10^−4^	DM	LP/VUS	P/VUS	50	29.5
*PCSK9*	rs185392267	chr1:55043921	C	T	p.Arg96cys	3	1.02 × 10^−4^	DM	P/LP/VUS	P/VUS	100	24.1
*PCSK9*	rs373323910	chr1:55061437	C	T	p.Arg582*	2	6.82 × 10^−5^	NR	LB	NR	50	34
***APOE***	**NR**	**chr19:44909102**	**G**	**A**	**p.Arg269His**	**1**	**3.41 × 10** ^**−5**^	**NR**	**NR**	**NR**	**100**	**24.8**

Alt, alternative allele; CADD, combined annotation dependent depletion; DM, disease-causing mutation; DM?, likely disease-causing mutation; FH, familial hypercholesterolemia; Het, heterozygous; HGMD, Human Gene Mutation Database; LP, likely pathogenic; LOVD, Leiden Open Variation Database; MAF, minor allele frequency; NR, not previously reported; P, pathogenic; Ref, reference allele; VUS, variant of uncertain significance; *HC, high cholesterol (refer to methods for details). Genomic positions are in reference to GRCh38 genome build, and protein positions are in reference to the NCBI sequence NP_000518.1. Bold indicates novel variants not reported in genetic variation databases.

### Comparing Familial Hypercholesterolemia Classification Based on Phenotypic and Genotypic Profiles of Study Subjects

**FIGURE 3:**
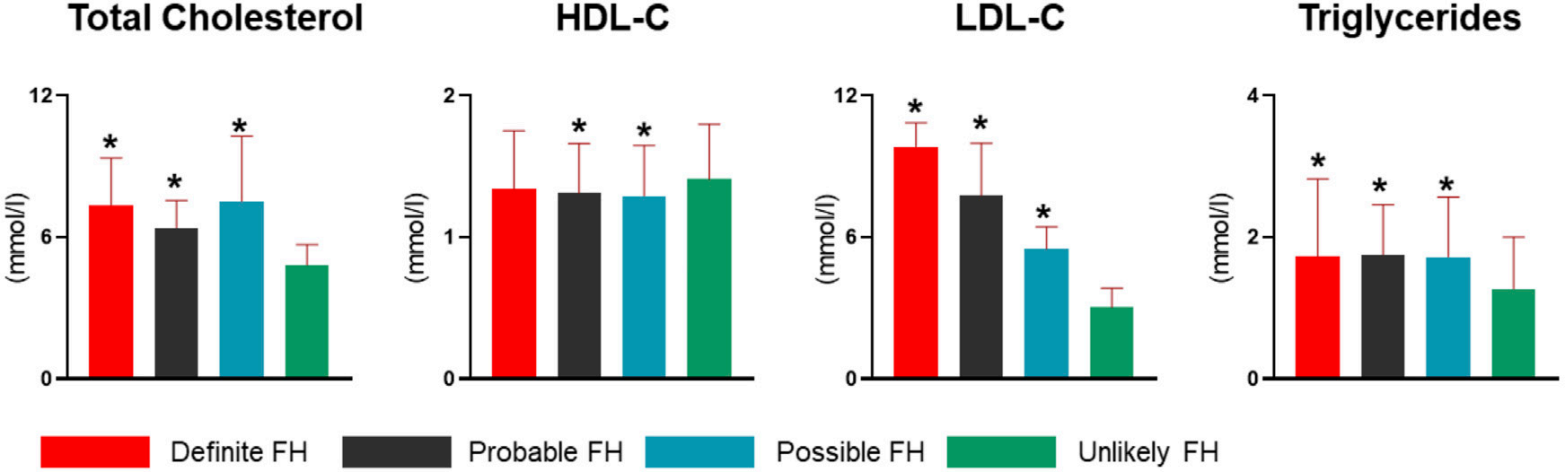
Lipid profiles of FH definition groups in the study cohort. Study subjects were categorized into definite, probable, possible and unlikely FH cases based on DLCN criteria, and using phenotype and genotype data. Bar charts represent levels of total cholesterol, HDL-C, LDL-C and triglycerides. LDL-C measurements were corrected for medication. Statistically significant (P < 0.05) differences among each FH definition compared to unlikely FH are denoted with an asterisk (*) in each plot.

Assessment of QBB study participants using DLCN criteria after adding information from genotypes and known FH-pathogenic mutations revealed 52 subjects as definite cases of FH (Table 4). Moreover, the probable, possible and unlikely FH cases classified based on combining phenotypic and genotypic evidence also showed some differences in numbers as opposed to classification based on FH-associated phenotypes only (Tables 1, 4). A number of probable FH (*n* = 8), possible FH (*n* = 14) and unlikely FH (*n* = 10) subjects were re-classified as definite FH because they were found to carry one of the known *pathogenic* mutations listed in Table 2. Additionally, 6 subjects who were originally classified as unlikely FH were re-classified as probable FH after considering genetic data. The proportions of smokers, myocardial infractions and family history of heart disease was considerably higher in definite cases of FH, while age at diagnosis of HC was significantly lower than those with unlikely FH (Table 4). Next, we investigated differences in lipid profile measurements of FH definition categorized by the DLCN criteria based on phenotypic and genotypic data (Figure 3). We found that total cholesterol, LDL-C and triglyceride levels were significantly higher in definite, probable and possible FH cases compared to subjects with unlikely FH (Figure 3). Moreover, HDL-C levels were significantly lower in probable and possible FH groups compared to unlikely FH.

**TABLE 4:** Characteristics of study subjects classified according to the Dutch Lipid Clinic Network (DLCN) criteria based on phenotypic and genotypic data.

Phenotypic trait(s)	Definite FH	Probable FH	Possible FH	Unlikely FH
Characteristic features				
n	52	57	627	12,942
Gender (males/females)	25/27	30/27	352/275	5,672/7,270
Age (years)	42.6 ± 13.2	46.5 ± 12.0*	46.6 ± 11.5*	39.7 ± 13.1
BMI (kg/m^2^)	29.6 ± 5.7	29.6 ± 5.7	30.1 ± 5.4*	29.5 ± 6.1
Smoker (%)	16 (30.8%)*	10 (17.5%)	139 (22.2%)*	2,169 (16.8%)
Medical history				
Hypertension (%)	9 (17.3%)	23 (40.3%)*	160 (25.5%)*	1984 (15.3%)
Diabetes mellitus (%)	17 (32.7%)*	26 (45.6%)*	195 (31.1%)*	2,562 (19.8%)
Self-reported hypercholesterolemia (HC)	42 (80.8%)*	51 (89.4%)*	455 (72.6%)*	3,519 (27.2%)
Age at HC diagnosis (years)	31.8 ± 10.9*	38.5 ± 9.9	39.8 ± 10.3	40.6 ± 10.6
Cholesterol-lowering medication	31 (59.6%)*	42 (73.7%)*	269 (42.9%)*	1,668 (12.9%)
History of myocardial infarction (MI)	2 (3.8%)*	1 (1.7%)	31 (4.9%)*	35 (0.3%)
Age at MI (years)	29.5 ± 9.5	53	44.6 ± 8.0	49.5 ± 14.9
History of angina	1 (1.9%)	0 (0.0%)	20 (3.2%)*	24 (0.2%)
Paternal heart disease (%)	28 (53.8%)*	15 (26.3%)	162 (25.8%)*	2,755 (21.3%)
Maternal heart disease (%)	9 (17.3%)	11 (19.3%)	108 (17.2%)*	1,615 (12.5%)
DLCN Score	11.8 ± 2.9*	6.9 ± 1.0*	3.5 ± 0.7*	0.4 ± 0.6

^†^Continuous traits are given as mean ± standard deviation from the mean. *Statistically significant (P < 0.05) compared to unlikely FH.

### Estimating the Prevalence of Familial Hypercholesterolemia in Qatar

**FIGURE 4:**
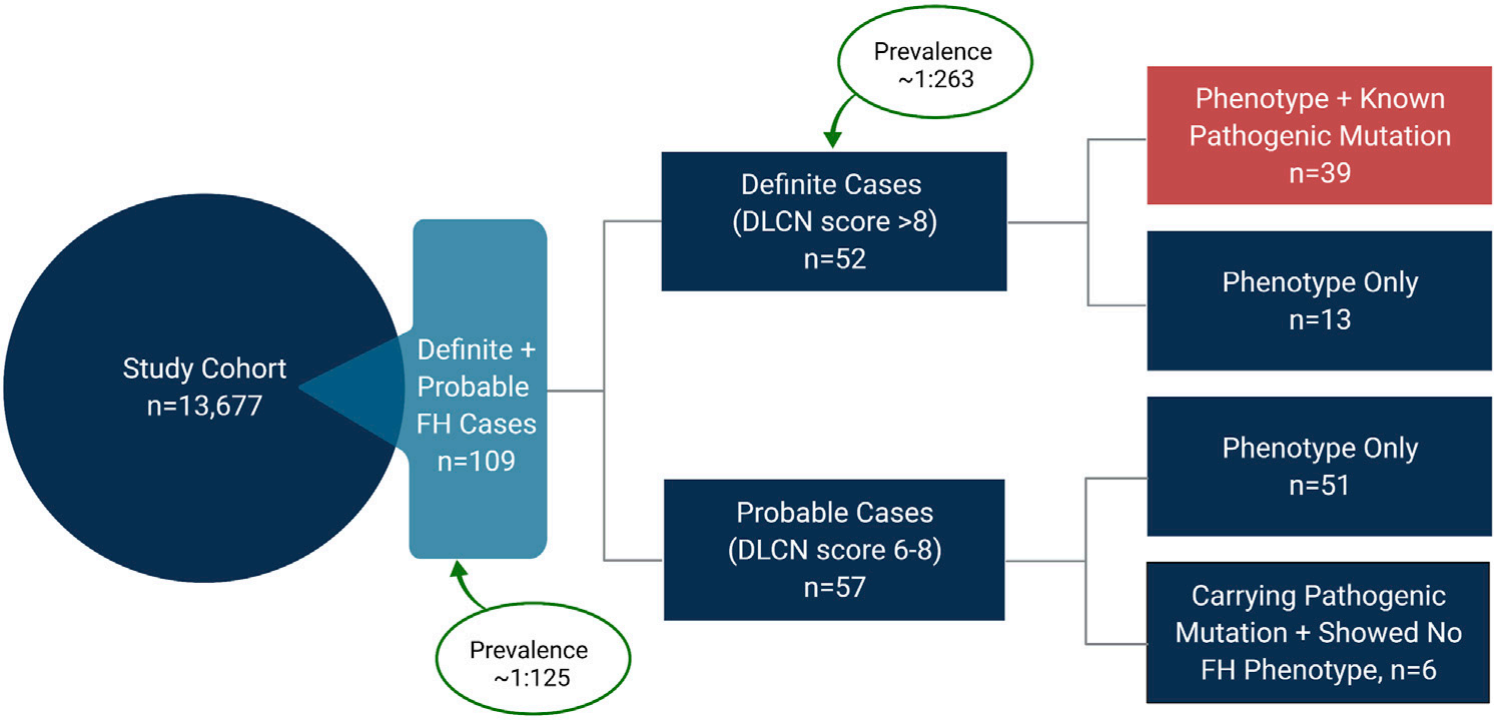
Prevalence of familial hypercholesterolemia (FH) in Qatar. The study cohort comprised 13,677 individuals. 109 subjects were identified as definite and probable cases of FH based on the Dutch Lipid Clinic Network (DLCN) criteria, yielding a prevalence of ∼1 in 125 in Qatar. 52 subjects were identified as definite cases of FH, indicating a prevalence of ∼1 in 263 for definite FH. Of the 52 definite cases, 39 subjects carried a known *pathogenic* mutation in *LDLR* or *PCSK9* and showed FH-related phenotypes. 57 subjects were identified as probable cases of FH with 51 subjects showing FH-related phenotypes (DLCN score 6–8), while 6 subjects carried a known *pathogenic* mutation (DLCN score 8), but their LDL-C levels were below the threshold of 4.0 mmol/L defined by DLCN criteria.

Of the total 13,677 subjects used in this study, we identified 109 as definite or probable cases of FH based on the fulfillment of the DLCN criteria (Figure 3). Based on this, the prevalence of FH in Qatar was therefore estimated at 0.8% (1 in 125). Notably, 39 subjects were diagnosed as definite cases of FH (DLCN score >8) based on genotypic mutation and phenotypic evidence, while additional 13 subjects showed a DLCN score >8 based on phenotype alone and did not carry any of the known *pathogenic* FH variants. Combined these definite FH cases accounted for 52 subjects and yielded an overall prevalence of 0.38% (∼1 in 263) for definite FH (Figure 4). In contrast, the number of subjects with probable FH (DLCN score ranging between 6–8), who carried a known *pathogenic* mutation but showed no phenotypic evidence, was 6, while 51 subjects were classified as probable FH based on phenotype. These subjects accounted for approximately half of the total suspected FH cases in our cohort. Of note, the prevalence of *possibly pathogenic* variants was 1:351 and the overall prevalence of FH in Qatar would be considerably higher (∼1 in 92) when these mutations are included in the estimation of the prevalence. However, due to the lack of evidence on the pathogenicity of these variants, further investigations are required to incorporate them in the assessment of population-based FH prevalence. In addition, we did not detect any homozygous FH or autosomal recessive mutations in our study cohort, indicating their low prevalence in our study population.

## Discussion

This study provides the first reliable estimate of the prevalence of FH in the Qatari population and presents a comprehensive survey of population-specific monogenic FH variants in a considerably large population-based cohort. Combining FH-related phenotypes with whole-genome sequence data revealed a prevalence of definite and probable FH cases in Qatar of ∼1 in 125, which puts Qatari subjects at a higher risk of FH than the global average of 1:250, previously determined from a meta-analysis of 19 published studies (Akioyamen et al., 2017), but at a moderately lower risk than neighboring countries in the Arabian peninsula (1:112) (Alhabib et al., 2021). The higher prevalence than global estimates could be attributed to the relatively consanguineous nature of the populations in the region and in Qatar, but the prevalence is considerably lower than those reported for populations with founder effects such as the African Ashkenazi and Lebanese (Seftel et al., 1989; Abifadel et al., 2009). In addition, we also did not detect any cases of HoFH, indicating its rare prevalence in Qatar.

We identified 17 mutations, characterized in the literature as *pathogenic*, out of which 16 were located in *LDLR*. Notably, 6 of these variants in *LDLR* were previously reported in ∼6000 subjects from Qatar but without any phenotypic associations with FH (Elfatih et al., 2021). In contrast, some of these 16 variants we identified have been directly associated with FH phenotypes in other populations. The p.Asp90Gly mutation in *LDLR* was reported in phenotypic FH patients from Western Australia who were screened for *LDLR*, *APOB* and *PCSK9* mutations (Hooper et al., 2012), while p.Glu31Lys has been reported in Asian Indian FH patients (Setia et al., 2020) and p.Phe282Leu was observed in Czech probands suspected to have FH (Tichý et al., 2012). Additionally, data from 6 studies investigating the influence of genotypes on response to PCSK9-targeting monoclonal antibody alirocumab also reported *LDLR* pGly382Val among the mutations identified in 898 HC patients (Defesche et al., 2004). While these mutations have been linked with FH, some of these variants are associated with severe disease complications related to FH. For instance, Cui *et al.* reported *LDLR* p.Arg81Cys mutation in patients with premature myocardial infarction (Cui et al., 2019).

Subjects carrying any of the known *pathogenic* mutations and with the presentation of FH-related phenotypes were identified as definite FH according to the DLCN. Notably, the definite FH identified in our study comprised a significantly higher proportion of subjects with self-reported HC and more importantly, a higher proportion of myocardial infarction and those diagnosed with HC at a younger age compared to subjects classified as unlikely FH. Moreover, a family history of heart disease was also considerably higher in definite FH cases. These clinical manifestations and prerequisites for FH classifications supported their classification into definite cases of FH. Considering the genotypic aberrations, the most frequently occurring variant was rs1064793799 found in 13 Qatari FH subjects, suspected to cause abnormal gene splicing. Single base changes occurring near or within introns can lead to intron retention, exon skipping, or activation of cryptic splice sites (Bourbon et al., 2009). Notably, an extreme reduction in *LDLR* expression, recorded in two families of Arab descent, was attributed to the activation of a cryptic splice acceptor site in *LDLR* due to a single substitution mutation (Shawar et al., 2012), however, this mutation was not detected in our study. In contrast, the majority of the mutations we detected in *LDLR* corresponded to missense mutations, which may be detected via PCR-based genotyping protocols for FH detection. For instance, Pandey et al. (2016) validated the detection of mutations in *APOB* and *LDLR*, by PCR, including p.Asp492Asn, also detected in our cohort. We also detected a *pathogenic* variant in *PCSK9* (p.Gly516Val) in two subjects with definite FH. This variant was detected in South African FH patients and was found to be pathogenic by functional studies (Huijgen et al., 2021).

Our study highlighted the importance of genetic testing to confirm FH diagnosis since only 19 subjects were classified as definite FH based on phenotype data while additional 33 subjects were considered definite FH when genetic testing was taken into consideration.

We also detected several *possibly pathogenic* variants in *LDLR, APOB, PCSK9* and *APOE* in our study subjects which were not previously confirmed in the literature as disease-causing mutations. These variants, however, were predicted to be among the top 1% of most deleterious mutations in the human genome and showed high penetrance for HC in our study. These variants could be presented as novel variants related to FH, however, further functional investigations are warranted for their validation. Notably, all variants detected in *APOB* were point mutations that could lead to defects in lipid hemostasis. Point mutations in *APOB* have been reported to cause FH by affecting its affinity for the LDL-R, which causes disruptions in LDL clearance via LDL-R-mediated internalization (Sharifi et al., 2017). These mutations differ from truncation mutations in *APOB* associated with hypobetalipoproteinemia. However, further functional investigations are warranted to confirm their pathogenicity in FH. Notably, five of the *possibly pathogenic* variants were novel and appear to be predominant in the Qatari population. Novel Qatari-predominant loci have been identified for many clinically relevant traits in a recent GWAS study of the QBB study participants (Thareja et al., 2021). A gene-based burden test for the *pathogenic* and *possibly pathogenic* variants in *LDLR*, *PCSK9* and *APOB* confirmed the association of these variants with HC. However, the gene-based burden test was not significant for *APOE* possibly because of the small number of *pathogenic* variants detected in our cohort. Overall, our findings reiterate that mutations in *LDLR* are the most common cause of FH in the Qatari population.

In this study, a substantially large cohort of 13,677 was used to achieve a reliable estimate for the prevalence of definite and probable cases of FH in the Qatari population, which was approximated to 1 in 125. The prevalence of subjects with *possibly pathogenic* variants was 1:351 and if future functional studies confirm the pathogenicity of these variants, the overall prevalence of FH will be 1:92 which is higher than our original estimate of 1:125 based on known *pathogenic* variants. We did not detect any cases of HoFH in our study cohort which indicates that HoFH is extremely rare in Qatar, as estimated in many European populations, but a larger sample size would be required to accurately estimate the prevalence of HoFH. However, one key limitation of our data collection was the lack of phenotypic data related to xanthomas and arcus cornealis for subjects or their first-degree relatives in our cohort. In addition, we did not investigate structural variants in FH-related genes, however, FH is predominantly caused by point mutations and a small fraction of FH cases are attributed to structural variants in FH-related genes. Overall, we detected 17 known *pathogenic* mutations in FH subjects and identified further 18 *possibly pathogenic* variants as novel candidates for FH. However, further functional investigations are warranted to investigate their pathogenicity. Overall, the clinical translation of FH-related variants reported herein may be explored further to design FH diagnostic tools.

## Data Availability

The data analyzed in this study is subject to the following licenses/restrictions: The raw whole-genome sequence data from Qatar Biobank are protected and are not available due to data privacy laws. Access to QBB/QGP phenotype and whole-genome sequence data can be obtained through an ISO-certified protocol, which involves submitting a project request at https://www.qatarbiobank.org.qa/research/how-apply, subject to approval by the Institutional Review Board of the QBB. Requests to access these datasets should be directed to https://www.qatarbiobank.org.qa/research/how-apply.
